# Frequency of Short- vs Long-Term Reporting of Bariatric Surgery Outcomes

**DOI:** 10.1007/s11695-022-06360-x

**Published:** 2022-11-24

**Authors:** Krishna K. Oochit, Safwan Shahwan, James Hughes, Georgios Kourounis

**Affiliations:** 1 Faculty of Medicine, University of Glasgow, Glasgow, G12 8QQ UK; 2 Department of Otolaryngology, Queen Elizabeth University Hospital, Glasgow, G51 4TF UK; 3 Department of General Surgery, James Cook University Hospital, Middlesbrough, TS4 3BW UK; 4 Department of Upper Gastrointestinal and Bariatric Surgery, South Tyneside & Sunderland NHS Foundation Trusts, Sunderland Royal Hospital, Sunderland, SR4 7TP UK

**Keywords:** Bariatric surgery, Follow-up, Outcomes

## Abstract

**Background and Aims:**

Bariatric surgery is an effective treatment for obesity. Though both short- and long-term outcomes have been reported, most of the published literature reports on short-term outcomes. Identification of post-operative weight regain and re-emergence of comorbidities requires medium- and long-term follow-up. We aimed to identify the distribution of follow-up times within the literature.

**Methods:**

We screened through 1807 articles from 9 PubMed Indexed bariatric surgery journals published between January to June of 2015 and 2021 and selected articles reporting weight loss as a main outcome. Follow-up intervals were defined as per American Society for Metabolic and Bariatric Surgery (ASMBS) guidelines.

**Results:**

Fifty-three and sixty-three articles were identified in 2015 and 2021 respectively. Reported follow-up lengths in 2015 were 60% short-, 26% medium-, and 14% long-term; while in 2021, there were 65% short-, 10% medium-, and 25% long-term articles. Of the articles reporting long-term outcomes in 2015 and 2021, 48%, and 70% of the included patients respectively had > 5 years follow-up.

**Conclusion:**

Though reporting of long-term outcomes increased, most published outcomes remain short-term. The UK National Bariatric Surgery Registry is helping to mitigate this. An increased effort and emphasis on reporting long-term outcomes is needed.

**Graphical Abstract:**

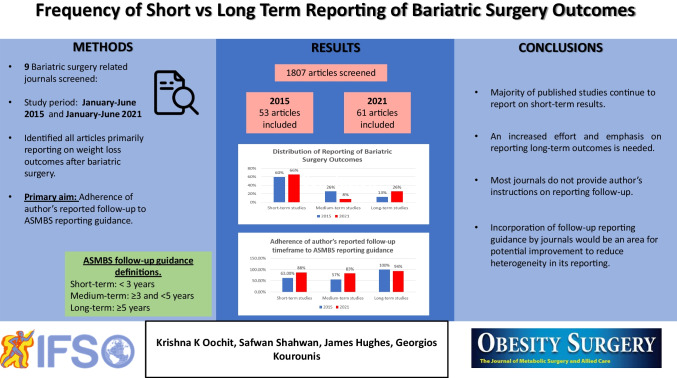

## Introduction

Obesity is a chronic disorder with multiple health implications. For the last few decades, it has become a growing epidemic with the worldwide prevalence nearly tripling between 1975 and 2016 [1]. Since its advent in the 1960s, bariatric surgery has gained popularity after demonstrating superiority over medical treatment for obesity [2, 3]. In 2019 alone, a total of 833,000 bariatric surgical operations were performed worldwide; a jump of more than 600,000 surgeries compared to the previous decade (2008–2009) [4]. Bariatric surgery has also been helpful in treating comorbidities associated with obesity including reducing cardiovascular deaths, as well as remission of type 2 diabetes mellitus and non-alcoholic fatty liver disease [5–8].

Evidence suggests that weight loss peaks at around 6 to 12 months postoperatively followed by weight loss stabilisation at around 18–24 months [9–13]. Some studies have reported 25–50% of their patients experiencing some weight regain after the 2-year mark following surgery [14–18]. The impact of weight regain and the re-emergence of comorbidities warrants long-term follow-up. While we have witnessed a surge in the bariatric surgery literature, the majority reports on short-term postoperative outcomes.

The heterogeneous nature of postoperative outcomes reported in bariatric surgery has already been raised. Inconsistent definitions of both follow-up intervals and weight loss outcomes greatly hinders data synthesis and analysis [19]. In 2015, the American Society of Metabolic and Bariatric Surgery (ASMBS) highlighted this and outlined clear reporting guidelines to address the need for standardisation of reporting bariatric surgery follow-up intervals and outcomes [20].

The primary aim of this study was to identify the prevalence of studies reporting on short-, medium- and long-term weight loss outcomes following bariatric surgery before and after the introduction of the 2015 ASMBS reporting guidance. Secondary aims included the adherence of authors’ reported follow-up timeframe to ASMBS reporting guidance, and outline attrition and follow-up rates in medium- and long-term follow-up publications.

## Methods

Journals indexed on PubMed with a focus on obesity and bariatric surgery were identified. These included Surgery for Obesity and Related Diseases, Obesity Surgery, Bariatric Surgical Practice and Patient Care, Clinical Obesity, Obesity Research & Clinical Practice, International Journal of Obesity, Obesity, Current Obesity Reports, and Journal of Obesity. All articles published within these journals in the first 6 months of 2015 (pre-ASMBS guidance) and 2021 (post-ASMBS guidance) were screened. Articles primarily reporting on weight loss outcomes following bariatric surgery were included. Animal studies, case-reports, editorials, reviews, or meta-analysis were excluded.

Two independent reviewers screened through all the articles to extract data from the eligible articles. When the two reviewers could not reach a consensus, a third one was consulted. Data collected from the articles included authors, type of study (prospective or retrospective), follow-up duration, the percentage of patients who completed each follow-up period, and follow-up rate at last study endpoint.

Follow-up duration was reported in one of three ways: mean follow-up length, median follow-up length, and longest follow-up. In articles where both mean follow-up and maximum follow-up were reported, the mean follow length was recorded. The collected data was categorised for further analysis into short-term (< 3 years), medium-term (≥ 3 and < 5 years), and long-term (≥ 5 years) as per ASMBS reporting guidance. [20]

Articles reporting follow-up outcomes from two different surgical groups or comparing groups undergoing two or more bariatric surgical procedures where the follow-up period for each of the cohorts were different were considered separate cohorts to conduct an appropriate analysis.

Statistical analyses were performed using SPSS Statistics for Windows version 27.0 (IBM, Armonk, NY). Categorical variables were described as numbers and percentages. They were compared using the Pearson χ^2^ analysis. Differences were considered of statistical significance if they reached a *p* < 0.05.

## Results

### Follow-Up Intervals

A total of 1807 articles were identified from our literature search. Fifty-three of 808 and 61 of 999 articles met our inclusion criteria in 2015 and 2021 respectively. In 2015, there were 21 (40%) prospective and 32 (60%) retrospective articles. In 2021, there were 22 (36%) prospective and 39 (64%) retrospective articles. Reported follow-up lengths among the 2015 and 2021 articles according to the ASMBS criteria are outlined in Table 1.

**Table 1:** Reporting on follow-up timeframes as per ASMBS guidance. Comparing number of articles reporting short-, medium-, and long-term outcomes in 2015 and 2021 as defined by the 2015 ASMBS guidelines

Follow-up	2015	2021	*p*
Short-term	32 (60%)	41 (65%)	0.601
Medium-term	14 (26%)	6 (10%)	0.016
Long-term	7 (14%)	16 (25%)	0.101

### Adherence of Author’s Reported Follow-Up Timeframe to ASMBS Reporting Guidance

**Fig. 1:**
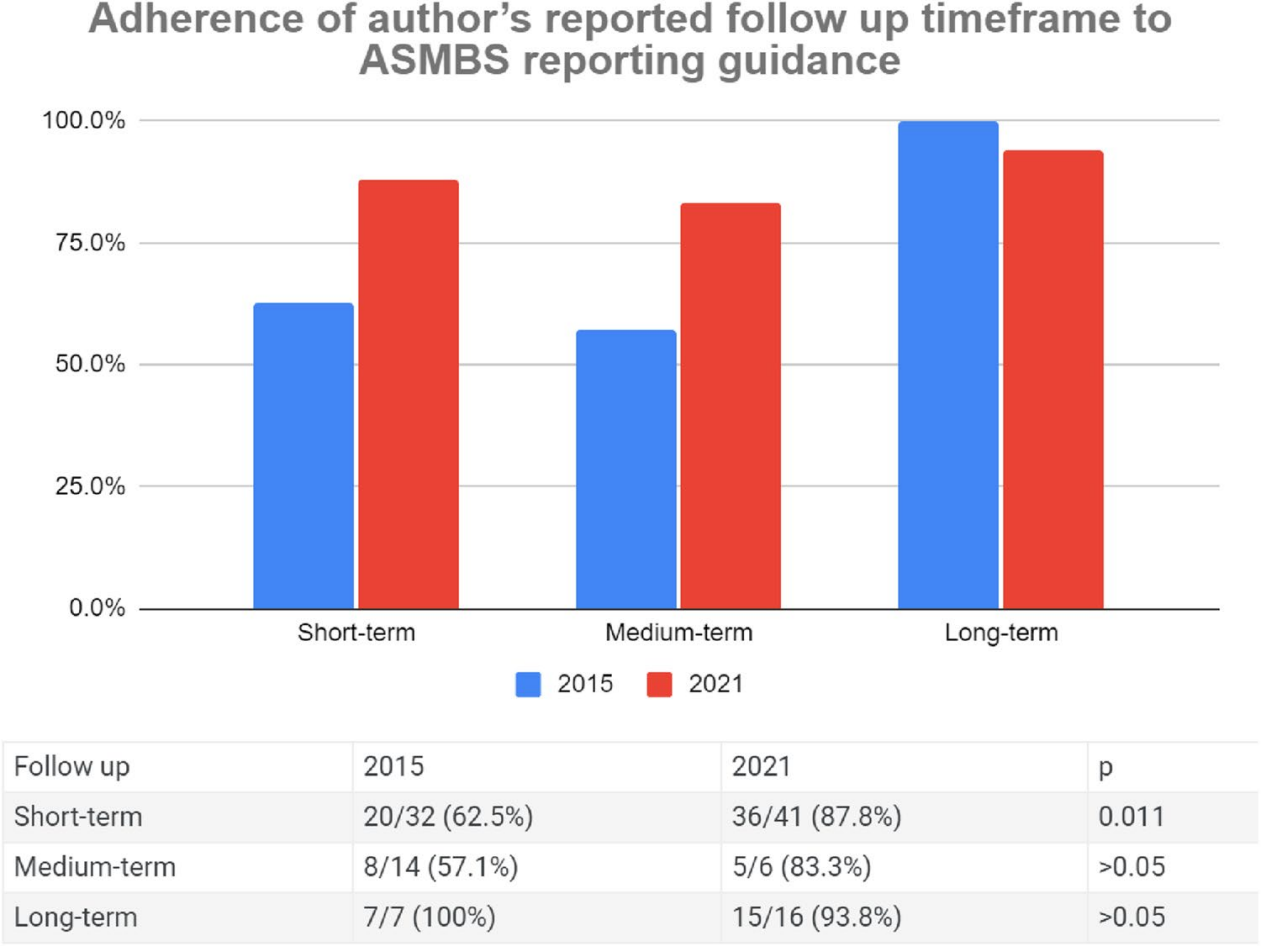
Adherence of author’s reported follow-up timeframe to ASMBS reporting guidance

Out of the 32 articles in 2015 that were classified as short-term under ASMBS follow-up reporting guidance, 20 (63%) reported their follow-up as short-term, 8 (25%) did not clearly specify their outcomes as short-term, 3 (9%) reported their outcomes as medium-term, and 1 (3%) reported their outcome as long-term. Adherence to the guidance increased in 2021 where 36/41 (88%) articles reported their outcomes as short-term, 4/41 (10%) did not specify, and 1/41 (2%) reported their outcomes as medium-term (Fig. 1).

Among the 14 articles that were classified as medium-term under the guidance in 2015, 8 (57%) reported their outcomes as medium-term, 2 (14%) did not specify, 3 (21%) reported their outcomes as long-term, and 1 (7%) reported their outcome as short-term. This improved in 2021 where 5/6 (83%) reported their outcomes as medium and 1/6 (17%) did not clearly specify (Fig. 1).

In 2015, all 7 articles that were classified as long-term under ASMBS reported their outcomes as long-term compared to 2021 where 15/16 (94%) reported their outcomes as long-term and 1/16 (6%) did not specify (Fig. 1).

### Reporting of Attrition and Follow-Up Rates

Among the short-term articles in 2015, 23/32 (72%) reported their attrition rates compared to 35/41 (85%) in 2021. All 14 articles (100%) and 4 out of 6 (67%) of the medium-term articles in 2015 and 2021 reported their attrition rates respectively. The mean follow-up rate at 3 years was 51% in 2015 compared to 84% in 2021. All the long-term articles in 2015 (*n* = 7) and 2021 (*n* = 16) reported their attrition rates. The follow-up rate at 5 years was 58% in 2015 in contrast to 70% in 2021. Table 2 outlines the results in full detail.

**Table 2:** Publications specifying attrition and follow-up rates in medium- and long-term follow-up publications. Comparing the number of articles reporting attrition rates in 2015 and 2021. Among the medium-term and long-term articles, the follow-up rates at 3, 4, 5, 10, and 15 years following surgery are reported in the table. In 2015, there were no papers reporting outcomes at 10 and 15 years

	2015	2021
Reported attrition rates		
Short-term	23/32 (72%)	35/41 (85%)
Medium-term	14/14 (100%)	4/6 (67%)
Long-term	7/7 (100%)	16/16 (100%)
Medium-term follow-up rates		
@ 3 years	51%	84%
@ 4 years	33%	21%
Long-term follow-up rates		
@ 5 years	58%	70%
@ 10 years	-	52%
@ 15 years	-	63%

## Discussion

Although the number of articles reporting long-term outcomes increased from 14% in 2015 to 25% in 2021, the majority has remained short-term. Studies have shown that short-term and long-term outcomes following bariatric surgery are different [10, 11]. It is mostly beyond the 2-year period after surgery that factors like weight loss stabilisation, weight loss failure, weight regain, or re-emergence of comorbidities become apparent [10, 11].

Publication bias has been raised as a possibility surrounding the lack of studies reporting long-term outcomes [19]. Short-term studies tend to over-inflate weight loss while masking the factors discussed above that would otherwise be exposed with longer follow-up duration. With the rise in numbers of different bariatric surgical techniques, long-term outcomes are crucial in identifying the long-term effects of these.

While we have noticed an improvement in the reporting standards of follow-up among articles in 2021 as per the ASMBS guidelines, inconsistencies still exist in the reporting of follow-up length and follow-up rate. Most articles have reported follow-up rates for their final endpoints only. As per the ASMBS reporting guidelines, it is still important to report the percentage follow-up at all the study endpoints as it serves both as a measure of the effectiveness of a follow-up programme and gives a truthful indication of the overall success rate of the operation [20].

Heterogeneity in reporting weight loss outcomes is a comparable significant issue in the bariatric surgical literature. A recent study found an increasing number of unique weight loss outcomes used in the literature between 2015 and 2021, clearly demonstrating the diversity that exists [21]. The uniform and in-depth reporting of follow-up data along with homogeneous reporting of weight loss outcomes is necessary to allow robust synthesis and meta-analyses among studies.

Furthermore, we identified that only one of the nine journals that were screened had specified follow-up reporting guidelines, including reporting the follow-up percentage at various study endpoints. Incorporation of clear follow-up reporting as a quality assessment criterion and in journals’ submission guidelines is an area for potential improvement which will help homogenise outcome reporting and limit any bias introduced by incomplete follow-up.

Another issue revolves around follow-up rates in the bariatric surgery literature. Studies by Fewtrell et al. and Kristman et al. showed that the ideal follow-up rate of any original cohort should be ≥ 80% [22, 23]. However, this is rarely achieved, even among the most cited bariatric literature [5]. Various reasons exist as to why patients drop-out from weight loss studies, with the major causes being weight loss failure, patient choice, or death. Suter et al. and Riele et al. found that patients who complied with follow-up have better weight loss outcomes following bariatric surgery compared to those who were lost to follow-up [24, 25]. Minimising the rate of patients who are lost to follow-up may therefore demonstrate improved patient outcomes. We do acknowledge, however, that there are multiple pragmatic barriers to maintaining long-term patient follow-up.

Bariatric surgery registries such as the UK National Bariatric Surgery Registry [26] are helping to prospectively collect data and mitigate the potential barriers that lead to insufficient reporting of long-term outcomes after bariatric surgery. This will be beneficial in informing both surgeons and patients regarding which procedure may suit them best.

We acknowledge certain limitations of our study. The search was conducted at two time-points which provided a cross-sectional snapshot as opposed to the trend over a period of time. To our knowledge, this is the first study investigating the frequency of short- and long-term outcomes after bariatric surgery and highlighting the heterogeneity in its reporting. The heterogeneity in follow-up length reporting as mean/median/maximum follow-up may have affected the categorisation process into short-, medium-, and long-term studies as articles reporting only their maximum follow-up may overestimate their whole cohort follow-up length compared to articles reporting their mean or median values. In cases where both were available, we used the mean/median values to classify articles.

## Conclusion

Obesity is a chronic disease requiring lifelong care. Though we welcome the increase in the number of articles that report long-term results, a major proportion remains to focus on short-term results. This limits the ability to extract meaningful data and sequential analyses. Long-term studies are crucial to clarify true and accurate outcomes following bariatric surgery and in turn generate robust evidence to base clinical decisions regarding life-changing surgeries. Incorporation of clear follow-up and attrition rates reporting guidance by journals for articles to adhere to is an area for potential improvement.

## Data Availability

Data available on request from the authors.
